# The gastric ecosystem: assessing resident microbiome vs. transient microorganisms in obesity and following bariatric interventions

**DOI:** 10.1038/s41522-026-01058-9

**Published:** 2026-06-16

**Authors:** Maxime Amoyel, André Bado, Tigran Poghosyan, Tiphaine Le Roy, Maude Le Gall

**Affiliations:** 1 Service d’Hépato-Gastro-Entérologie, Hôpital Bichat-Claude-Bernard, Assistance Publique-Hôpitaux de Paris, Université Paris Cité, Paris, France; 2 Inserm Université Paris Cité UMRS 1149 Centre de Recherche sur l’Inflammation Paris, Paris, France; 3 Service de Chirurgie Digestive, Oesogastrique et Bariatrique, Hôpital Bichat-Claude-Bernard, Assistance Publique-Hôpitaux de Paris, Université Paris Cité, Paris, France; 4 Sorbonne Université, Inserm, Nutrition and Obesities: Systemic Approaches Nutriomics, Paris, France

**Keywords:** Diseases, Gastroenterology, Microbiology

## Abstract

Long considered a sterile environment due to its acidity, the stomach is increasingly recognized as a low-biomass but structured microbial ecosystem. Advances in culture-independent sequencing reveal a diverse gastric microbiome shaped by acid secretion, mucosal physiology, diet, medication use, and *Helicobacter pylori* colonization, although distinguishing metabolically active residents from transient oropharyngeal or dietary microorganisms remains a significant challenge. In healthy individuals, gastric mucosal communities primarily consist of Bacillota, Pseudomonadota, Actinomycetota, Bacteroidota, and Fusobacteriota, with genera such as *Streptococcus*, *Prevotella*, *Rothia*, and *Veillonella* consistently detected. Obesity is associated with altered gastric physiology, including higher pH, delayed emptying, and increased bile reflux, alongside distinct microbial signatures. *Helicobacter pylori* remains a dominant ecological factor modifying acidity, inflammation, and niche structure, while proton-pump inhibitors significantly “oralize” the gastric microbiome. Although animal models suggest that bariatric surgery can remodel the gastric microbiome, no human study has yet characterized gastric microbial changes following sleeve gastrectomy, gastric bypass, or endoscopic bariatric therapies, leaving the translational relevance of preclinical findings uncertain. Given the central role of the stomach in nutrient sensing and metabolic hormone secretion, its microbial ecosystem warrants dedicated investigation.

## Introduction

For decades, the human stomach was regarded as a nearly sterile organ, its acidic environment presumed to preclude microbial survival^1^. This paradigm shifted with the discovery of *Helicobacter pylori*, which not only revolutionized the understanding of peptic ulcer disease and gastric carcinogenesis but also revealed that the stomach environment could sustain microbial life^2^. Beyond *H. pylori*, recent advances in high-throughput DNA sequencing have revealed the presence of a large number of bacterial taxa in the stomach, suggesting the existence of a diverse and complex gastric microbiome. Indeed, bacteria from multiple phyla, including Bacillota, Pseudomonadota, Actinomycetota and Bacteroidota (formerly Firmicutes, Proteobacteria, Actinobacteria, and Bacteroidetes phyla, respectively) are consistently detected in gastric samples^3^. These microbes appear to play roles in maintaining mucosal homeostasis and may modulate gastric physiology, acid secretion, and local immune responses^4^.

It is important to note that the detection of microbial DNA in the stomach does not necessarily indicate the presence of a stable, metabolically active microbiota. Sequencing approaches cannot distinguish between viable and non-viable microorganisms nor identify their ecological origin, and gastric microbial signatures likely reflect a mixture of resident bacteria, transient oral or dietary microorganisms, or microbes refluxing from the proximal intestine. Beyond *H. pylori*, evidence for bacterial viability in the gastric environment remains limited, although selected taxa such as *Lactobacillus* and *Fusobacterium* genera have been shown to survive under acidic conditions^5,6^. Nonetheless, the constant detection of consistently structured microbial communities in the stomach in association with health and disease states suggests that alterations of the gastric microbiome could extend beyond classical infectious or inflammatory diseases^4,7,8^. In particular, metabolic disorders such as obesity and the physiological changes induced by bariatric surgery weight-loss procedures may profoundly affect gastric ecology^9^. These shifts could, in turn, influence nutrient processing, hormone secretion, and even systemic metabolic outcomes.

Beyond its digestive functions, the stomach plays a central role in metabolic regulation and body-weight control, notably through the secretion of key gastrointestinal hormones such as ghrelin and GLP-1, which influence appetite, energy balance, and glucose homeostasis^10^.

The present review aims to summarize current knowledge of the gastric microbiome, describe the methodological and analytical challenges inherent to its study, and discuss how surgical, endoscopic, and pharmacological interventions, including bariatric and anti-obesity treatments, may modulate this microbial ecosystem.

## Methods for studying the gastric microbiome

### Sampling strategies

The accurate characterization of the gastric microbiome largely depends on the method and site of sample collection. Gastric biopsies remain the gold standard, as they allow the study of mucosa-associated communities directly interacting with the epithelial surface. Nevertheless, luminal bacteria may transiently contact the mucosal surface without true mucosal adherence and be collected with biopsies, potentially confounding the interpretation of mucosa-associated microbial profiles. Alternatively, gastric juice samples capture luminal microorganisms transiently exposed to the acidic environment, while mucosal brushing techniques can provide a broader representation of the adherent microbiota with minimal tissue injury. The stomach itself is not homogeneous: microbial distribution varies across anatomical regions such as the fundus, corpus, and antrum, likely reflecting local differences in pH, mucus composition, and glandular cell types. Although regional differences in microbial distribution across fundus, corpus, and antrum are biologically plausible, available human data do not demonstrate major compositional differences between antrum and corpus in healthy individuals. Vasapolli et al.^11^ and Wurm et al.^12^ both reported broadly similar microbial profiles between these two regions using 16S rRNA gene amplicon sequencing of mucosal biopsies, suggesting that macroscopic anatomical differences may not translate into large-scale microbiota divergence under physiological conditions. In contrast, *H. pylori* colonization, tends to exhibit region-specific patterns, predominantly antrum-based in early infection, extending to pangastric distribution with disease progression, which may impose localized ecological effects that may be missed by single-site sampling. Notably, no study has yet investigated region-specific gastric microbiota changes following bariatric surgery, representing a critical knowledge gap given the profound anatomical remodeling induced by these procedures. Because of the anatomical continuity with the oral cavity and the duodenum, avoiding contamination by oral, pharyngeal and duodenal microbiota is essential. Strict sterile techniques, the use of negative procedural controls, and sequencing of potential contaminant sources (saliva, endoscope channel washings) are recommended to ensure data reliability^5,13,14^. These approaches mainly characterize the fasting gastric microbiota, while postprandial microbial dynamics remain poorly characterized and would likely require capsule-based sampling technologies, which are still in development. In preclinical models, gastric sampling approaches differ substantially from those used in humans. Rodent studies typically rely on gastric lavage, tissue scraping, or full mucosal dissection rather than targeted endoscopic biopsies, thereby limiting anatomical precision and increasing the risk of luminal contamination. Moreover, the obligate coprophagic behavior of rodents partially homogenizes microbial communities across gastrointestinal compartments through continuous fecal-oral cycling. This fundamental difference complicates the interpretation of compartment-specific findings and must be carefully considered when extrapolating preclinical gastric microbiome data to human biology.

### Sequencing and analytical approaches

The study of gastric microbial diversity has evolved from culture-based methods to culture-independent molecular techniques. In 1997, partial 16S rRNA gene sequencing was reported by Liu et al.^15^ and still remains the most widely used approach. This technique, referred to as 16S rRNA gene amplicon sequencing, is based on PCR amplification of hypervariable regions of the bacterial 16S rRNA gene, enabling taxonomic identification flanked by conserved subregions. This technique is cost-effective, sensitive, and well-suited to low-biomass samples such as gastric biopsies; however, its resolution is limited, typically at the genus level, and it provides no functional information, while remaining prone to PCR-related biases^16,17^.

To overcome these constraints, shotgun metagenomic sequencing offers an untargeted whole-genome strategy capable of characterizing microbial communities at species or strain level and identifying functional pathways relevant to gastric ecology, such as urease activity, oxidative stress responses, or bile acid metabolism. This deeper resolution and extended taxonomic coverage makes shotgun metagenomics a powerful tool, but it is significantly more expensive and technically demanding, and its application to gastric samples is complicated by the high proportion of host DNA, which can dominate sequencing reads and limit microbial signal detection^18,19^. Despite these constraints, a small but growing number of studies^20–22^ have applied shotgun metagenomics specifically to gastric samples and have contributed functional metagenomic data from the gastric environment, identifying pathways related to urease activity, oxidative stress responses, bile acid metabolism, and virulence-associated functions that are not detectable by 16S rRNA gene amplicon sequencing alone. Together, these studies illustrate both the potential of shotgun metagenomics in the gastric niche and its current scarcity, underscoring the need for larger dedicated studies using optimized host-depletion strategies. Moreover, in low-biomass gastric samples, shotgun metagenomics often provides lower effective microbial sequencing depth than targeted 16S rRNA gene sequencing approaches due to the absence of amplification steps specifically targeting microbial DNA. An alternative approach to enrich for metabolically active bacteria consists of 16S rRNA transcript amplicon sequencing, which involves reverse transcription of ribosomal RNA into cDNA prior to PCR amplification. Since rRNA abundance broadly correlates with cellular metabolic activity, this strategy may provide a more dynamic view of the viable gastric microbiota compared to DNA-based methods. However, the technique is technically demanding and remains prone to RNA degradation in the acidic gastric environment, limiting its current application in gastric microbiome research. Importantly, while 16S rRNA transcript-based sequencing can identify potentially viable and metabolically active bacteria, a significant step forward compared to DNA-based approaches, it does not provide information on which genes these bacteria are actually expressing. Characterizing microbial gene expression in the stomach would require bacterial RNA sequencing (RNAseq), which is technically even more demanding than 16S rRNA cDNA amplicon sequencing, particularly given the low bacterial biomass, the high proportion of host RNA, and the chemical instability of RNA in the acidic gastric environment.

Beyond DNA sequencing-based methods, emerging multi-omic approaches such as metatranscriptomics, metaproteomics, metabolomics, and culturomics provide additional layers of insight. Metatranscriptomics captures actively expressed microbial genes, offering a dynamic view of metabolic activity, although the RNA instability noted above limits its application in the gastric niche. Metaproteomics characterizes microbial proteins directly, providing complementary functional insight that is less susceptible to RNA degradation artifacts, although gastric studies remain scarce. Metabolomics enables the profiling of bioactive molecules produced by host–microbe interactions, while culturomics uses high-throughput culture methods to isolate viable strains, thus allowing to decipher metabolically active cells from transient dead microorganisms. However, culturomics is inherently biased toward organisms capable of growth conditions implemented in each study, including aerobic versus anaerobic atmospheric requirements, culture media composition, and incubation parameters, and may systematically under-represent strict anaerobes, fastidious taxa and microorganisms relying on cross-feeding with other taxa that are nonetheless present and potentially active within the gastric niche. Together, these approaches provide complementary perspectives, and their integration in multi-omic frameworks represents a next step toward a comprehensive understanding of the gastric microbiome’s structure and function^23^.

### Specific challenges of gastric samples

The stomach poses unique methodological challenges for microbiome research. Its low microbial biomass makes sequencing results highly susceptible to contamination from reagents or the environment, amplifying batch effects and potentially generating false-positive signals. Rigorous experimental design, including blank extraction and sequencing controls, is therefore indispensable. Computational tools such as decontam or ALDEx2 and SCRuB, a source-tracking-based decontamination method particularly suited to low-biomass environments, can help distinguish true biological signals from background noise^14,24–26^.

Additionally, the acidic environment and enzymatic activity can lead to partial DNA degradation, reducing yield and sequence quality. This necessitates optimized DNA extraction protocols and careful interpretation of low-abundance taxa. Together, these challenges highlight the need for standardized workflows and stringent quality control to ensure robust and reproducible analysis of the gastric microbiome^14,24,27^.

## The gastric microbiome in health

### Composition in healthy individuals

For many years, research on the gastric microbiota remained largely neglected, owing to the long-standing dogma that the stomach’s strong acidity rendered it sterile and inhospitable to microbial life^1^. In addition to the acidic milieu, other physicochemical factors such as bile reflux, the thickness of the mucus layer^28^, and efficient gastric peristalsis were believed to further impede microbial colonization. Moreover, dietary nitrates converted to nitrites and subsequently to nitric oxide within the stomach were thought to contribute additional antimicrobial pressure^29^. Together with the technical difficulties of sampling and the absence of sensitive detection methods, these factors perpetuated the misconception of a sterile gastric cavity.

This view was overturned in 1982 with the discovery of *Campylobacter pyloridis*, later renamed *Helicobacter pylori*, by Warren and Marshall^2^. The identification of *H. pylori*, belonging to the Pseudomonadota phylum, revealed that specific bacteria could indeed colonize the gastric mucosa, resist acid through urease activity, and induce chronic inflammation leading to gastritis, peptic ulcer, and, in a minority of cases, gastric cancer. Around the same period, culture-based studies also detected acid-resistant species belonging to *Streptococcus*, *Neisseria*, and *Lactobacillus* genera, suggesting that the gastric environment could harbor a broader range of microbial life^30–34^. These organisms may originate from the oropharyngeal cavity or the duodenum through swallowing or reflux. While many are transient, evidence indicates that some may be stably established^5,6^.

Subsequent molecular investigations confirmed that the stomach hosts a diverse and complex microbial community. Early 16S rRNA gene-based studies by Bik et al.^13^ and Li et al.^35^ identified over one hundred non-*H. pylori* phylotypes distributed among five dominant phyla: Bacillota, Pseudomonadota, Actinomycetota, Bacteroidota and Fusobacteriota (formerly Firmicutes, Proteobacteria, Actinobacteria, Bacteroidetes, and Fusobacteria). At the genus level, *Streptococcus*, *Prevotella*, *Rothia*, *Veillonella*, and *Haemophilus* were consistently detected in large relative abundance in healthy individuals. Two recent studies by Zhou^36,37^ demonstrated that the presence of *Streptococcus anginosus* and *Streptococcus constellatus* within the gastric mucosa is associated with an increased risk of gastric cancer, further supporting the notion that a true gastric microbiome exists. Later work using pyrosequencing and next-generation methods confirmed this pattern and highlighted a surprisingly consistent microbial structure across populations of different geographic and ethnic backgrounds, albeit with considerable interindividual variability^5,38^.

Importantly, the stomach displays several distinct microbial profiles. Mucosal samples are typically enriched in Bacillota and Pseudomonadota, whereas Actinomycetota and Bacteroidota dominate the gastric juice (luminal compartment), an observation underscoring the complementary value of sampling both mucosal and luminal compartments to achieve an accurate representation of gastric microbial profiles. Despite the acidic and dynamic environment, these microbial communities are forming part of a balanced ecosystem that may contribute to mucosal homeostasis, nitric oxide metabolism, and local immune regulation.

Overall, the healthy gastric microbiome can be described as a low-biomass but structured community that appears to be shaped by host physiology and environmental exposure. Its stability depends on gastric acidity, mucosal integrity, and host–microbe interactions. Understanding this baseline composition is essential for interpreting how diseases, medications, or bariatric interventions alter this ecosystem.

### Environmental and host factors

The composition of the gastric microbiota is dynamic and modulated by multiple endogenous and external factors, including gastric acidity, diet, medication use, inflammation, and microbial interactions. Among these, *H. pylori* colonization and proton pump inhibitor (PPI) therapy are the two most influential determinants of microbial structure in the stomach.

### *Helicobacter pylori* colonization

*H. pylori* remains the most extensively studied gastric bacterium and is a key driver of ecological change within the stomach. Chronic colonization induces mucosal inflammation, epithelial remodeling, and progressive glandular atrophy, which collectively lead to hypochlorhydria and a rise in gastric pH^39,40^. This altered environment allows usually transient microorganisms to persist and colonize the mucosa. Animal and human studies have shown that *H. pylori*-positive individuals exhibit an increased relative abundance of Pseudomonadota, Spirochaetota, and Acidobacteriota, with a concomitant reduction of Actinomycetota, Bacteroidota, and Bacillota phyla compared with uninfected subjects. Conversely, *H. pylori*-negative stomachs tend to be dominated by *Streptococcus* and *Prevotella* genera^41^. Beyond direct ecological competition, *H. pylori* also produces ammonia and bicarbonate from urea hydrolysis, creating localized niches of neutral pH that may favor the growth of cohabiting species. Thus, this bacterium indirectly modifies the gastric habitat, possibly facilitating the survival of acid-sensitive taxa such as *Streptococcus*, *Prevotella*, and certain members of the Bacteroidota phylum, which are less adapted to the low-pH gastric environment and are consistently enriched in H. pylori-positive individuals^41–44^. Nonetheless, studies using 16S rRNA gene sequencing, including the seminal work of Bik et al.^13^ and Khosravi et al.^45^ found no major shift in overall community diversity between *H. pylori*-positive and -negative individuals, suggesting a subtler, localized and context-dependent influence. The impact of *H. pylori* likely depends on infection duration, degree of mucosal atrophy, and the presence of premalignant lesions.

### Proton pump inhibitors (PPIs) and acid suppression

Among pharmacological agents, PPIs exert the most profound impact on the gastric microbiota. By markedly reducing acid secretion, PPIs increase gastric pH above 4, a threshold that enables bacterial overgrowth and “oralization” of the gastric environment. Several studies have demonstrated that long-term proton pump inhibitor use induces a shift toward an oral-type microbial profile, characterized by enrichment of *Streptococcus* and *Rothia* and a relative loss of typical acid-adapted gastric taxa, including *Helicobacter*, specific Pseudomonadota, and mucosa-associated Actinomycetota. Importantly, PPI-induced changes may affect both the relative proportions of bacterial taxa and the overall gastric bacterial load, reflecting true bacterial overgrowth rather than compositional shifts alone. These changes are driven by gastric acid suppression and elevation of intragastric pH. Even short-term PPI exposure has been shown to induce measurable microbial alterations and bacterial overgrowth^46–50^.

This drug-induced ecological change has clinical implications, as it may promote small intestinal bacterial overgrowth (SIBO)^51^, modulate gastric immune signaling, and confound microbiome analyses in clinical studies. For this reason, PPI exposure represents a critical confounding factor that must be carefully controlled or reported in all gastric microbiome investigations.

### Other potential influencing factors

Even if there is no data, potential additional modulators can be supposed, such as dietary composition, which can transiently affect the abundance of Lactobacilli and other fermentative bacteria; antibiotic exposure, which induces long-term depletion of beneficial taxa and expansion of opportunists; and gastric motility or bile reflux, which alter the luminal flow and chemical environment. Host genetics and mucosal immune status may also contribute to interindividual variability.

## The gastric microbiome in obesity

The association between the intestinal microbiota and obesity has been documented, with microbial shifts influencing nutrient absorption, lipid metabolism, and systemic inflammation. Of note, most published studies examining gut microbiota in obesity rely primarily on fecal samples, which more accurately reflect the colonic rather than the gastric microbial compartment. However, fewer studies have explored the gastric microbiota as a potential contributor to obesity-related pathophysiology.

The stomach represents the first point of contact between ingested nutrients and the gastrointestinal microbiome, and its bacterial composition may modulate digestion, hormone secretion, and metabolic signaling.

### Obesity and gastric inflammation

Obesity is characterized by significant physiological changes that may reshape the gastric ecosystem. Several studies have shown that gastric pH is higher^52^, gastric emptying is accelerated^53^, and duodenogastric bile reflux is more frequent in individuals with obesity^54,55^. Beyond physiological alterations, obesity is often associated with distinct dietary patterns, including differences in meal composition, eating frequency, and fasting duration, which may modulate gastric microbial dynamics, although this hypothesis has yet to be formally investigated. These factors, combined with increased intra-abdominal pressure and systemic low-grade inflammation, may weaken mucosal defenses and modify the physicochemical environment of the stomach. Clinically, obesity has been associated with a higher prevalence of non-atrophic gastritis and mucosal inflammation, independent of *H. pylori* infection, suggesting that altered gastric physiology and inflammation could contribute to microbiota remodeling. It is also suggested that *H. pylori* infection is more common in people with obesity suggesting that obesity could be a risk factor^56–59^.

### Experimental evidence from animal models

In a pivotal murine study, He et al.^60^ showed that a high-fat diet (HFD) induces early alterations of the gastric mucosal microbiota that precede changes observed in the fecal microbiota. After 12 weeks of HFD, mice exhibited reduced microbial diversity (lower Shannon and Chao1 indices), with increased Bacillota and Pseudomonadota, and decreased Bacteroidota and Verrucomicrobiota, including depletion of the beneficial microbe *Akkermansia muciniphila*. These microbial shifts were associated with the onset of metabolic disorders such as insulin resistance and dyslipidemia, suggesting that gastric dysbiosis may act as an early metabolic driver in obesity. Notably, a similar depletion of *A. muciniphila* was observed in fecal samples from the same animals, suggesting that the gastric microbiota changes reported may partly reflect coprophagia-mediated dissemination of intestinal microbial shifts rather than independent gastric dysbiosis.

A second murine study by Arita and Inagaki-Ohara^61^ further extended these findings by demonstrating that as little as 1 week of HFD feeding is sufficient to induce drastic changes in gastric microbiome composition in C57BL/6J mice, characterized by marked *Lactobacillus* dominance and complete disappearance of *Bifidobacterium pseudolongum*, preceding the development of intestinal metaplasia, precancerous lesions of the gastric mucosa. Crucially, this study identified gastric leptin receptor (Lepr) signaling as a key mechanistic link between HFD, gastric microbiome alterations, and mucosal pathogenesis. To dissociate local gastric effects from potential systemic or central influences of leptin, the authors generated gastrointestinal epithelium-specific Lepr conditional knockout mice (T3b-Lepr cKO), in which Lepr is selectively deleted in the gastrointestinal epithelium while central leptin signaling and body weight regulation remain intact. These mice did not develop spontaneous obesity and were protected against both HFD-induced gastric dysbiosis and intestinal metaplasia, supporting the conclusion that gastric epithelial Lepr signaling, rather than systemic or hypothalamic effects, drives the observed microbial and mucosal changes. Conversely, oral gavage of gastric contents from HFD-fed donors into antibiotic-pretreated wild-type recipients partially reproduced metaplastic changes, despite a standard chow diet. These findings suggest that functional host–microbiome interactions at the gastric level, mediated by leptin signaling, may drive early precancerous mucosal remodeling in the context of diet-induced obesity, an observation that extends well beyond simple compositional dysbiosis.

These results should be interpreted cautiously, given the coprophagic behavior of rodents, which partially homogenizes gastrointestinal microbial communities along the gut.

### Human studies of gastric microbiota in obesity

Recent human data support the existence of obesity-specific gastric microbial signatures. In a recent comparative study, Gazi et al.^9^ analyzed gastric tissue samples from 19 patients with obesity undergoing bariatric surgery and 18 normal-weight individuals with functional dyspepsia using 16S rRNA gene sequencing. While alpha diversity (Shannon and Simpson indices) did not differ significantly between groups, beta diversity analyses revealed distinct microbial profiles in subjects with obesity. The Bacillota/Bacteroidota ratio was significantly higher in the group with obesity, accompanied by lower abundances of Bacteroidota and Fusobacteriota. At the genus level, 15 taxa differed significantly, with *Prevotella_7*, *Veillonella*, *Cupriavidus*, and *Acinetobacter* among the most represented (>3% relative abundance). These findings suggest that, although global diversity may remain stable, obesity is linked to compositional shifts in the gastric ecosystem, possibly reflecting changes in pH, motility, and bile reflux. However, it remains difficult to determine whether these compositional changes are specific to the gastric niche or partly driven by obesity-related alterations of the oral microbiota, which may secondarily influence gastric microbial profiles due to the constant saliva flow^62^.

Further evidence comes from Gutiérrez-Repiso et al.^63^, who characterized the gastric microbiota of 41 patients with morbid obesity undergoing sleeve gastrectomy. In *H. pylori*-negative, PPI-free subjects, the gastric mucosa was dominated by Bacillota (40%), Bacteroidota (23%), and Pseudomonadota (22%), with *Streptococcus*, *Bacteroides*, and *Prevotella* as predominant genera. Importantly and surprisingly, both PPI use and *H. pylori* infection significantly reduced bacterial diversity and altered microbial composition, correlating with less favorable metabolic outcomes one year after surgery, such as reduced weight loss and higher fasting glucose.

Complementing these findings, Martínez-Montoro et al.^64^ performed 16S rRNA gene sequencing of gastric biopsies taken during sleeve gastrectomy from adults with obesity *H. pylori* (HP) negative and lean controls. HP negative individuals with obesity exhibited lower microbial richness and distinct taxonomic and functional signatures, characterized by an enrichment of Bacillota, notably *Streptococcaceae*, and Pseudomonadota, alongside decreased Bacteroidota and Actinomycetota. Functional prediction analyses revealed increased metabolic pathways related to lipid metabolism, oxidative stress, and inflammation, supporting a potential mechanistic link between gastric dysbiosis and metabolic impairment.

Overall, these studies demonstrate that obesity is associated with gastric microbial dysbiosis, featuring an increased Bacillota/Bacteroidota ratio, reduced diversity, and enrichment in oral-like taxa such as *Streptococcus* and *Veillonella*. Both clinical and experimental data suggest that alterations in the gastric ecosystem may arise early in the metabolic disease process, potentially contributing to impaired hormonal signaling, inflammation, and nutrient handling. Although causality remains to be established, the stomach, traditionally viewed as an environment hostile to microbes, is emerging as a dynamic microbial niche that may influence systemic metabolic health.

## Impact of bariatric surgery on the gastric microbiome

Bariatric surgery profoundly alters gastrointestinal anatomy and physiology, reshaping the microbial ecosystem of the stomach and small intestine. These changes include increased gastric pH, altered motility, reduced gastric volume, bile acid reflux, and exposure of distal gut microbiota to undigested nutrients. Collectively, these modifications create new ecological niches that lead to major microbial shifts, both at the gastric and intestinal levels, which may contribute to the metabolic benefits observed post-surgery^65,66^.

### Gastric microbiota alterations after bariatric surgery

#### Laparoscopic sleeve gastrectomy

Laparoscopic sleeve gastrectomy (LSG) involves removing roughly 70–80% of the stomach along the greater curvature, creating a tubular gastric conduit. This restrictive procedure reduces gastric volume, accelerates emptying, and alters acid secretion, resulting in significant hormonal changes such as decreased ghrelin levels. Clinically, LSG leads to substantial and durable weight loss, with an average excess weight loss of 50–70% within the first 2 years^67^.

To date, no human study has directly characterized gastric microbiota changes after sleeve gastrectomy, and evidence is limited to animal models. A recent high-fat diet–induced obesity study in rats by Park et al.^68^ demonstrated that sleeve gastrectomy leads to significant restructuring of the gastric microbial ecosystem. Using 16S rRNA gene sequencing of gastric tissue, the authors reported clear postoperative shifts in beta diversity, along with marked changes in the relative abundance of several taxa, including increased *Streptococcus* and reduced *Lactobacillus* and *Romboutsia*. These microbial modifications occurred alongside improvements in metabolic parameters (fasting glucose, leptin, HDL-cholesterol), suggesting a potential interaction between gastric microbial remodeling and metabolic recovery after surgery. Although these findings provide valuable mechanistic insight, their relevance to humans remains uncertain, as rodents are physiologically coprophagic, a behavior that partially homogenizes gastrointestinal microbial communities of the different gut compartments. Consequently, extrapolation of murine gastric microbiome data to humans should be made with caution, and robust clinical studies are still lacking to determine whether similar gastric microbial changes occur following bariatric surgery. Nonetheless, these experimental observations show that bariatric surgery may remodel the gastric microbiome in ways that could influence host metabolic signaling, potentially through interactions with gastric hormones and inflammatory mediators.

Endoscopic sleeve gastroplasty (ESG) is an emerging minimally invasive bariatric procedure that reduces gastric volume through endoscopic full-thickness suturing, without gastric resection. ESG induces significant weight loss and metabolic improvement, while preserving gastric anatomy and mucosal continuity^69,70^. However, to date, no study has evaluated the impact of ESG on the gastric microbiome, despite profound changes in gastric volume, motility, and intragastric pressure. This lack of data highlights the need for dedicated studies exploring how endoscopic gastric remodeling may influence gastric microbial ecology.

### Roux-en-Y gastric bypass

Roux-en-Y gastric bypass (RYGB) is a surgical technique combining restriction and malabsorption by creating a small gastric pouch connected to the jejunum, bypassing the distal stomach and duodenum. This rearrangement modifies nutrient flow and enhances incretin responses, which, in addition to reduced dietary intake, result in rapid and sustained weight loss (over 60–75% of excess weight loss), as well as significant metabolic improvements, particularly in people with type 2 diabetes^71^.

**Fig. 1:**
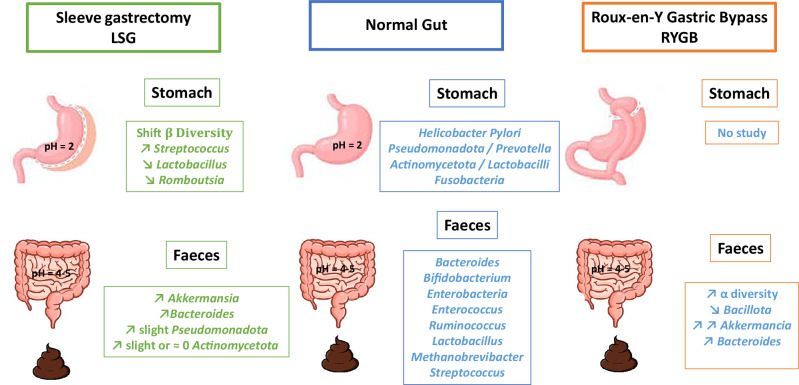
Comparative overview of gastric and fecal microbiota profiles in normal physiology and following bariatric surgery. This figure summarizes the main compositional changes reported in the gastric microbiota across physiological and pathological conditions. Human gastric microbiota data (blue) were obtained primarily from mucosal biopsy samples analyzed by 16S rRNA gene amplicon sequencing, unless otherwise specified. Data from animal models (green) (rodent, high-fat diet-induced obesity model; sleeve gastrectomy model) were obtained from mucosal tissue samples using 16S rRNA gene amplicon sequencing. Fecal microbiota profiles are based on published studies in human cohorts with obesity and following bariatric surgery. To date, no human postoperative gastric microbiota data are available; therefore, animal model findings are presented for illustrative mechanistic purposes only and should not be directly extrapolated to humans, given the coprophagic behavior of rodents. Abbreviations: LSG laparoscopic sleeve gastrectomy, RYGB Roux-en-Y gastric bypass.

Although Roux-en-Y gastric bypass (RYGB) profoundly modifies gastric anatomy, acid exposure, bile reflux, and nutrient flow, no study has yet characterized the gastric microbiota before and after these procedures. Existing work focuses almost exclusively on the fecal microbiome^72^, leaving the gastric niche entirely unexamined. This gap likely reflects technical limitations, including low bacterial biomass, risk of oral contamination, and difficulty obtaining repeated postoperative biopsies. Thus, how gastric microbial communities respond to bypass remains unknown, representing a major and unexplored research avenue with potential metabolic relevance. Figure 1 provides a comparative overview of gastric and fecal microbiota compositional changes reported across physiological conditions, obesity, and bariatric surgery, integrating both human and rodent data and highlighting the current absence of human postoperative gastric microbiota studies.

### Influence of *Helicobacter pylori* and PPI treatment

#### *Helicobacter pylori* and metabolic outcomes

Multiple studies have explored the interaction between *H. pylori* and bariatric surgery outcomes. Goday et al.^73^ in 2018, found that patients receiving *H. pylori* eradication therapy before LSG experienced greater short-term BMI reduction and improved triglyceride and glucose profiles compared to *H. pylori*-negative patients. Conversely, the same team in 2024 reported that *H. pylori*-treated patients had less weight loss and smaller glucose reductions 12 months after surgery, suggesting that eradication and associated antibiotic therapy may transiently disrupt the gastric and intestinal microbiota^74^.

A 2025 meta-analysis^75^ confirmed that *H. pylori*-positive status increases the risk of postoperative complications (odds ratio for ulcer = 6.88, CI 5.60–8.45) and supports routine preoperative eradication.

### Proton Pump Inhibitors (PPIs)

PPIs are frequently prescribed postoperatively and significantly alter gastric microbial composition. Gutiérrez-Repiso et al.^63^ demonstrated that both *H. pylori* infection and PPI therapy reduce bacterial diversity and modify predicted metabolic functions in the gastric mucosa of LSG patients. These changes correlated with poorer weight loss and glucose control 12 months after surgery, suggesting that altered gastric microbial ecology may influence postoperative metabolic response. However, as both PPIs and, to a lesser extent, *H. pylori*, also induce microbial changes in more distal segments of the gastrointestinal tract, including the fecal microbiota, it remains difficult to attribute these metabolic differences specifically to gastric microbial alterations alone.

Altogether, these studies underscore the importance of accounting for *H. pylori* status and PPI exposure when assessing post-bariatric microbial and metabolic changes.

## Discussion

The gastric microbiome, once considered negligible due to the stomach’s acidic environment, has emerged as a complex and dynamic microbial niche shaped by host physiology, diet, medication, and *H. pylori* colonization. The role of *H. pylori* in the development of gastritis and peptic ulcers, and its contribution to adenocarcinoma, has been the matter of extensive studies and is presently well known.

Current evidence demonstrates that obesity is associated with altered gastric physiology, such as higher pH, accelerated emptying, and increased bile reflux, which may contribute to specific microbial signatures distinct from those observed in lean individuals. Preliminary data in humans and experimental models suggest that obesity-related changes in gastric ecology could participate in metabolic dysfunction, although causality remains unproven.

Despite the growing interest in gut microbial ecosystems, the gastric microbial communities remain profoundly understudied. Human data investigating the impact of bariatric surgery on the gastric microbiome are missing. While sleeve gastrectomy and gastric bypass profoundly remodel gastrointestinal anatomy and physiology and are known to influence the intestinal microbiome, no study has yet characterized their effect on the gastric mucosa or gastric juice microbiota. Available evidence is limited to animal models or preoperative analyses in bariatric candidates, with no longitudinal human dataset exploring postoperative gastric microbial dynamics.

Similarly, pharmacological anti-obesity agents and endoscopic bariatric procedures, like endoscopic sleeve gastroplasty, have not been evaluated in relation to the gastric microbiome. GLP-1 plays a central role in postprandial metabolic regulation, and its secretion is profoundly altered by bariatric surgery, particularly RYGB, which markedly increases postprandial GLP-1 levels through accelerated nutrient delivery to the distal gut. Importantly, the gastric mucosa itself harbors GLP-1-expressing cells, the density of which increases following bariatric surgery^10^. A growing body of evidence suggests that GLP-1 receptor agonists modify gut microbial composition, diversity, and richness^76^, potentially through effects on gastric motility, acid secretion, and gastric emptying. Whether these pharmacological and surgical GLP-1-related changes extend to the gastric microbial ecosystem has not been studied and represents a relevant question for future investigation, given the role of the stomach in GLP-1 biology after bariatric surgery. In addition, bariatric and endoscopic interventions profoundly modify eating behavior, meal timing, and gastric filling kinetics, factors that may independently shape gastric microbial signatures and warrant further investigation.

Importantly, future research should aim to better discriminate between viable, metabolically active microorganisms and transient or non-viable microbial components detected by sequencing approaches, as both may exert biological effects on gastric physiology and systemic metabolism.

Overall, the gastric microbiome represents an unexplored frontier with potential metabolic, inflammatory, and hormonal implications. Future studies integrating gastric sampling, multi-omic approaches, and longitudinal follow-up are needed to determine whether shifts in gastric microbial communities contribute to obesity pathophysiology or to the metabolic benefits of bariatric and endoscopic interventions. This gap offers an opportunity to advance our understanding of gastric biology and to explore potential therapeutic targets in obesity and metabolic disease.

## Data Availability

Data sharing is not applicable to this article as no datasets were generated or analyzed during the current study.
